# Modeling Human Neurological and Neurodegenerative Diseases: From Induced Pluripotent Stem Cells to Neuronal Differentiation and Its Applications in Neurotrauma

**DOI:** 10.3389/fnmol.2017.00050

**Published:** 2017-02-28

**Authors:** Hisham Bahmad, Ola Hadadeh, Farah Chamaa, Katia Cheaito, Batoul Darwish, Ahmad-Kareem Makkawi, Wassim Abou-Kheir

**Affiliations:** Department of Anatomy, Cell Biology and Physiological Sciences, Faculty of Medicine, American University of BeirutBeirut, Lebanon

**Keywords:** induced pluripotent stem cells (iPSCs), neuronal differentiation, Parkinson's disease (PD), Huntington's disease (HD), Amyotrophic lateral sclerosis (ALS), Alzheimer's disease (AD), spinal cord injuries (SCI)

## Abstract

With the help of several inducing factors, somatic cells can be reprogrammed to become induced pluripotent stem cell (iPSCs) lines. The success is in obtaining iPSCs almost identical to embryonic stem cells (ESCs), therefore various approaches have been tested and ultimately several ones have succeeded. The importance of these cells is in how they serve as models to unveil the molecular pathways and mechanisms underlying several human diseases, and also in its potential roles in the development of regenerative medicine. They further aid in the development of regenerative medicine, autologous cell therapy and drug or toxicity screening. Here, we provide a comprehensive overview of the recent development in the field of iPSCs research, specifically for modeling human neurological and neurodegenerative diseases, and its applications in neurotrauma. These are mainly characterized by progressive functional or structural neuronal loss rendering them extremely challenging to manage. Many of these diseases, including Parkinson's disease (PD), Huntington's disease (HD), Amyotrophic lateral sclerosis (ALS) and Alzheimer's disease (AD) have been explored *in vitro*. The main purpose is to generate patient-specific iPS cell lines from the somatic cells that carry mutations or genetic instabilities for the aim of studying their differentiation potential and behavior. This new technology will pave the way for future development in the field of stem cell research anticipating its use in clinical settings and in regenerative medicine in order to treat various human diseases, including neurological and neurodegenerative diseases.

## Introduction

Stem cell research is considered one of the most captivating areas of cell biology mainly due to the unique properties of stem cells and their potential use in cell-based therapies to treat a variety of diseases, including Parkinson's disease (PD), Alzheimer's diseases (AD), Diabetes Mellitus (DM), and many others (Correia et al., 2005; Pagliuca et al., 2014; Sproul, 2015). Given their unique regenerative abilities, these cells provide new potentials in the area of regenerative or reparative medicine.

Embryonic stem cells (ESCs), which are derived from the inner cell mass (ICM) of blastocysts, are pluripotent cells that have the ability to proliferate indefinitely. But still they maintain their pluripotency with the capability to differentiate into cells of all three germ layers: ectoderm, mesoderm and endoderm (Evans and Kaufman, 1981; Martin, 1981). In addition, these cells serve as an internal repair system, limitlessly regenerating into either differentiated cell progeny or additional stem cells (Keller, 1995; Thomson et al., 1998). Since first isolated in 1998, human ESCs have featured high importance as a potential treatment of a variety of diseases like PD, spinal cord injury (SCI) and DM (Thomson et al., 1998). However, the extraction of ESCs raises sharp ethical controversies as they are derived from human embryos and their transplantation in patients may present serious risks with a possibility of rejection (Lo and Parham, 2009).

Alternative approaches to the derivation of ESCs from the ICM of pre-implanted embryos are now available and these tend to avoid ethical issues. Such methodologies directly generate pluripotent stem cell lines from differentiated adult somatic tissue and include nuclear transfer, cell fusion or direct reprogramming (Hochedlinger and Jaenisch, 2006). In 2006, a landmark discovery was published by the Yamanaka group at Kyoto University as they induced the expression of only four pluripotency-associated transcription factors, Oct3/4, Sox2, c-Myc, and Klf4 (OKSM), in mouse fibroblast cells resulting in the generation of ESC-like cells, called induced pluripotent stem cells (iPSCs). These cells are similar to the ESCs in their morphology, gene expression, proliferation and teratoma formation (Hochedlinger and Jaenisch, 2006; Takahashi and Yamanaka, 2006; Takahashi et al., 2007; Wernig et al., 2007; Hadadeh et al., 2012). These iPSCs are now widely used for various applications, such as autologous cell therapy, monogenic and multigenic diseases modeling, and as substrates for drug, toxicity, differentiation and therapeutic screens.

Reprogramming highly depends on the efficient delivery and the suitable expression of certain factors into specific cell types, under particular culture conditions and within a period of time. Although direct reprogramming is a simple technique, it differs depending on the cell type, species and delivery method. It is rather a slow and vulnerable process that may be affected by several factors that hinder the efficiency, reproducibility and the quality of the resulting iPSCs. To date, the most popular donor somatic cells are fibroblasts, being used in more than 80% of all reprogramming experiments published (González et al., 2011). Yet, other cell types have been used in reprogramming such as human primary keratinocytes, cord blood CD133+ cells, and peripheral blood mononuclear cells (Aasen et al., 2008; Giorgetti et al., 2009; Su et al., 2016).

A successful management of the outcome of somatic cell reprograming to iPSCs is related to both the reprograming technique and the type of cells that are used. Here, it is important to note that although fibroblasts are the most widely used cells for iPSCs generation, this does not necessarily mean that they represent the highest efficacy. In fact, since the first approach toward creating iPSCs was obtained from fibroblasts (Takahashi and Yamanaka, 2006), subsequent protocols tried to reproduce that process before other alternatives for fibroblasts were investigated. Fibroblasts are easy to cultivate in culture and much is known about these cells in research. An important characteristic of these cells that makes them great candidates is the low methylation levels of the promotor regions of *OCT4* and *NANOG* that can be associated with the favorable reprogramming of the cells. Furthermore, fibroblasts can be easily obtained from patients through biopsy and are relatively inexpensive and widely commercially available by many companies. However, the fact that fibroblasts are highly proliferative poses few disadvantages as the non-programmed fibroblasts can have the opportunity to overgrow the existing reprogrammed cells and consume the growth factors in the media. This can usually be overcome by using a low passage not exceeding passage 5 in order to avoid accumulated genomic changes (Raab et al., 2014).

Reprogramming can be induced by the co-introduction of some genes that are expressed early during development, such as *OCT4, SOX2, NANOG, UTF1*, and *SALL4*, and which are implicated in the maintenance of the pluripotent potential of the ICM (Niwa, 2007; Zhao et al., 2008; Tsubooka et al., 2009). Supplementation with other genes such as *c-MYC, KLF4, TERT*, and *SV40LT* can enhance cell proliferation in a direct or indirect manner (Park et al., 2008b). Additionally, microRNAs (miRNAs) have been implicated in pluripotency and reprogramming, such as the miR-290 cluster and miR-302 cluster miRNAs (Wang et al., 2008; Mallanna and Rizzino, 2010). On the other hand, there are several chemical compounds that have proven to enhance reprogramming in different cell types. Those compounds are known to alter DNA methylation or cause chromatin modifications and they include DNA methyltransferase inhibitor 5′-azacytidine or histone deacetylase (HDAC) inhibitors (such as hydroxamic acid (SAHA), trichostatin A (TSA), and valproic acid (VPA)) (Huangfu et al., 2008). The delivery of the OKSM transcription factors into mouse or human fibroblasts is achieved using different viral and non-viral constructs, as well as integrative and non-integrative systems approaches, the latter of which have presented major problems for iPSCs generation. Four main groups of different non-integrative approaches are available: integration-defective viral delivery, episomal delivery, RNA delivery and protein delivery (González et al., 2011). There is no best reprogramming strategy that can be used to fit all purposes. The choice of the strategy highly depends on the purpose of the research; whether it focuses on understanding the mechanisms of reprogramming or on generating clinically relevant iPSCs. Integrative methods with lentiviruses can be sufficient for the former use while non-integrative approaches should be used for the latter to limit genomic modifications.

Understanding and treating many diseases have been constrained by the absence of *in vitro* models, especially because culturing primary cells affected by the diseases is very challenging. Limitations primarily lie in the access to patient's tissues as the priority goes for diagnosis, in addition to the complications in obtaining some cell types, such as neural or cardiac tissues, and to maintaining these cells *in vitro*. However, the development of stem cell studies and the novel discovery of iPSCs provided an important source of cells to conduct *in vitro* studies (Unternaehrer and Daley, 2011). Such establishment of human iPSCs (hiPSCs) has led to new clinical strategies for using them as universal sources in regeneration therapy of damaged organs and tissues (Pei et al., 2010). Moreover, iPSCs generated from a patient affected by a certain disease possibly reproduces the disease phenotype (Egashira et al., 2011). In view of this, different kinds of patient-specific iPSCs have been generated to model human neurodegenerative diseases, such as Parkinson's disease (PD) (Byers et al., 2012), Huntington's disease (HD) (Nekrasov et al., 2016), Amyotrophic lateral sclerosis (ALS) (Chestkov et al., 2014), and Alzheimer's disease (AD) (Mungenast et al., 2016).

## iPSCs and ectodermal differentiation

The ectoderm is the first germ layer to emerge during gastrulation, which is initiated by the formation of the primitive streak within the epiblast. Cell lineages derived from the ectoderm differentiate to form mainly the epidermis (including skin, hair, nails, and sweat and sebaceous cutaneous glands) and the nervous system (central and peripheral). The development of the vertebrate nervous system is shown to be regulated temporally and spatially by gradients of signaling molecules that may have either inhibitory or activating roles. These molecules are important for neuronal migration (Khodosevich and Monyer, 2011), axonal guidance and outgrowth (Chilton, 2006), interneuronal synapses (Scheiffele, 2003) and neuron-glia interaction (Fields and Stevens-Graham, 2002). Subsequently, experiments have demonstrated that this process is under the control of a combination of small-molecule endogenous inhibitors of bone morphogenic protein (BMP) and TGFβ/activin/nodal signaling (Morizane et al., 2011), which promote highly efficient neural induction from both human ESCs and iPSCs. Additionally, it was shown that DLK1 has a role in stimulating neurogenesis of human and mouse iPSC-derived neural progenitors via modulating Notch and BMP signaling (Surmacz et al., 2012). Using such small molecules to induce differentiation of iPSCs into a specified lineage shows a potent approach to generate specific cell types in order to better understand the biological function and disease processes, as well as to use these cells in drug screening and cell therapy.

## Use of iPSCs to model neurodegenerative diseases

Recently, efforts have been dedicated to generate defined lineages of neural cells from ESCs and iPSCs. These cells serve to better understand the molecular mechanisms underlying the pathophysiology of many intractable neurodegenerative diseases such as PD, HD, ALS, and AD aiming for the development of effective therapies. The present lack of precise models of these diseases conveys the significant discrepancies in understanding the mechanisms underlying their pathophysiology. In the last decade, the ability to reprogram somatic cells into iPSCs have enhanced the effectiveness of human *in vitro* models of neurological diseases (Mungenast et al., 2016). Moreover, the differentiation of these iPSCs into disease-relevant cell types have allowed comprehensive molecular analyses of multiple disease states. Indeed, neurons differentiated from patient-specific iPSCs provide a valuable tool to model specific molecular phenotypes of neurodegenerative diseases *in vitro* (Heman-Ackah et al., 2016). In this aspect, the introduction of human iPSCs with disease-specific genetic backgrounds requires precise and flexible genome engineering tools.

Among different groundbreaking experiments, the Clustered Regularly Interspaced Short Palindromic Repeats (CRISPR) endonuclease is considered the less cumbersome and the most flexible system to execute precise genome editing in human pluripotent stem cells (Kime et al., 2016). The genomic revolution made by this system and others, including the Zinc Finger Nucleases (ZFN) and Site specific nucleases (SPN), offered the simplest and most powerful approach toward manipulating the produced iPSCs for mimicking any disease and adding more advancement and efficiency to the resulting cells (Mandegar et al., 2016). Recently, Rubio et al. described a new platform where neurons can be generated *in vitro* and manipulated using CRISPR/Cas9 to inactivate specific genes associated with different neuropathologies in humans (Rubio et al., 2016). Moreover, in a recent study, it was shown that CRISPR can be used to exert precise alterations in the expression of the critical PD-related gene, *SNCA*, in human iPSC-derived neurons (Heman-Ackah et al., 2016). Remarkably, Paquet et al. (2016) established a procedure that allows the introduction of specific point mutations into iPSCs using CRISPR, thus generating human iPSCs with specific combinations of homozygous and heterozygous early-onset Alzheimer's-associated mutations. Certainly, the rapid development of iPSCs and genome-editing technologies are important tools for disease modeling that hold promise for applications in gene therapy.

**Figure 1:**
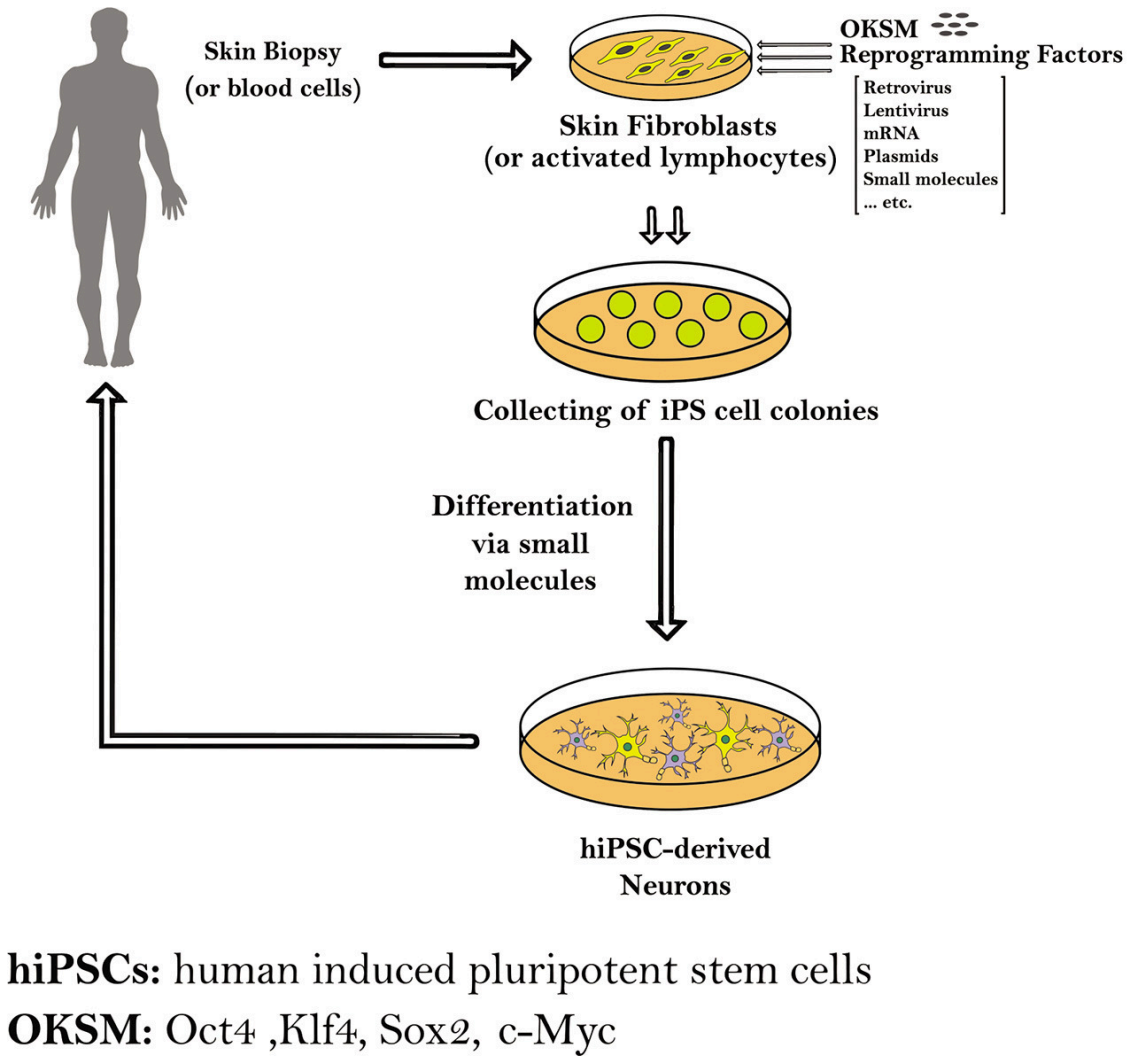
**Schematic diagram showing the methods used to generate induced pluripotent stem cells (iPSCs) from human somatic cells as skin fibroblasts or blood cells**. The hiPSCs derived from a patient carrying a certain genetic mutation in a neurodegenerative disease have the capacity to differentiate into different neurons. Those patient-specific hiPSCs and hiPS-derived neurons can be expanded and further differentiated into mature neural subtypes specific to certain neurodegenerative diseases.

In this review we will focus on the reprogramming of somatic cells into patient-specific hiPSCs to model four neurodegenerative diseases, namely Parkinson's disease (PD), Huntington's disease (HD), Amyotrophic lateral sclerosis (ALS) and Alzheimer's disease (AD), and other applications in neurotrauma, through summarizing the different protocols that are used in each for the reprogramming and differentiation processes (Figure 1).

### iPSCs and parkinson's disease

The study of Parkinson's disease (PD) is challenging due to the inaccessibility of affected human midbrain dopaminergic (mDA) neurons on which to base experimental research, and the rarity of animal models that follow the major disease characteristics. Despite this, it has been uncertain whether the involved mechanisms would also occur in human neurons affected by the disease. Consequently, molecular pathways underlying this pathology are still not well-defined. Although the majority of PD cases are sporadic, some rare familial forms of this disease have led to the discovery of PD-linked genes. This discovery was imperative for deciphering the cellular and molecular mechanisms of PD (Gasser, 2007; Schulz, 2008) and for creating transgenic animal and cellular models expressing these PD- associated genes. Recent development in PD research generated different neuronal cell types, which were previously inaccessible, by deriving PD-linked iPS cell lines that could be used for autologous transplantation (Park et al., 2008a; Soldner et al., 2009). Such approaches present exciting promises to elucidate the etiology of PD and develop novel potential therapeutics (Byers et al., 2012; Table 1).

**Table 1:** **Parkinson's disease modeled with patient-specific hiPSCs**.

**Authors**	**Donor somatic cell used**	**Generated iPSCs used**	**Neuronal differentiation method and results**
Soldner et al., 2009	Human skin fibroblasts	PD Patient-Derived hiPSCs	EB formation method in EB medium on non-adherent culture plates for 8 days, then selecting neural precursor cells and culturing them in ITS medium containing fibronectin, growth factors FGF2, FGF8, and SHH, followed by withdrawal of growth factors for 8 days to attain terminal differentiation
Cooper et al., 2010	Human skin fibroblasts	PD Patient-Derived hiPSC lines	Using a high activity form of SHH and FGF8a, rather than FGF8b, and specific regionalization by RA, directly from EB stage, to produce DA neurons with maintained stability defined by an expression marker code of FOXA2/TH/β-tubulin
Devine et al., 2011	Human skin fibroblasts	PD Patient-Derived hiPSC lines with triplication ofSNCA	Feeder-free floor plate induction, and dual SMAD inhibition for 1 day by Noggin, SB431542 and dorsomorphin, followed by SHH, WNT1 and DKK1 blocking antibody treatment for 8 days, then switching culture conditions to promote maturation of DA neurons
Sánchez-Danés et al., 2012	Epidermal keratinocytes and dermal fibroblasts	PD Patient-Derived hiPSC lines	Lentiviral vector-mediated engineering of hiPSCs to overexpress Lmx1a in neural progenitors in order to generate enriched populations of neurons with the characteristics of A9 ventral midbrain DA neurons

*PD, Parkinson's disease; hiPSCs, human induced pluripotent stem cells; EB, embryoid body; SHH, sonic hedgehog; RA, retinoic acid; DA, dopaminergic; Lmx1a, LIM homeobox transcription factor 1a; DKK1, Dickkopf WNT signaling pathway inhibitor 1; SNCA, α-synuclein gene*.

DA neurons that were first generated from mouse iPSCs in 2008 and transplanted into the striatum of a rat PD model, have shown to ameliorate functional deficits (Wernig et al., 2008). Recently, human fibroblasts have also been used to produce PD patient iPS-cell derived DA neurons. Soldner et al. (2009) was the first to report efficient reprogramming of human skin fibroblasts from 5 patients with sporadic PD into hiPSCs, and subsequent differentiation of these cells into DA neurons. Neural differentiation was first induced by embryoid body (EB) formation method in EB medium on non-adherent culture plates for 8 days, and then neural precursor cells were selected and cultured in ITS medium containing fibronectin, growth factors FGF2, FGF8, and sonic hedgehog (SHH). This was followed by withdrawal of growth factors for 8 days to attain terminal differentiation. Cells produced stained positive for tyrosine hydroxylase (TH) and neuron-specific class III-β-tubulin (TUJ1) confirming their DA neural nature (Soldner et al., 2009). Besides, the obtained hiPSCs, using the Cre-Recombinase excisable viruses, uniformly expressed the pluripotency markers Tra-1-60, SSEA4, OCT4, SOX2, and NANOG, in addition to possessing similar morphology to the human ESCs. Interestingly, the OCT4 promoter region of the obtained hiPSCs was in a hypomethylated state in contrast to the hypermethylated state which is found in the parental fibroblasts cells. There were also no differences in the ability or efficiency to differentiate dopaminergic cells from PD and non-PD patients (Soldner et al., 2009).

While sporadic PD cases are the most prevalent, and lack specific causative genes and a definite genetic basis, there still exists a barrier toward making any genotypic verification from the obtained differentiated cells (Park et al., 2008a). Previously, studies on PD animal models and ESC-derived dopaminergic transplantation has shown to be successful (Ganat et al., 2012; Grealish et al., 2014; Kang et al., 2014). Neuroprogenitor cells (NPCs) differentiated from iPSCs were transplanted in mouse fetal brain and were able to migrate into various regions of the brain, differentiate into both glia and neurons, and integrate into pre-existing brain network (Wernig et al., 2008). When neurons were transplanted, they exhibited a normal behavior and started branching and forming synapses. Eventually, they matured releasing dopamine and thus reducing the motor manifestations of PD in PD rat and monkey models (Wernig et al., 2008; Hallett et al., 2015; Han et al., 2015). Applying such cell therapy for PD in particular is very promising and is under a lot of extensive research for better optimization before reaching human clinical trials.

Although, the co-expression of TH and TUJ1 defines the stable phenotype of the produced DA neurons, studies have proven an additional role of the forkhead transcription factor, FoxA2 in maintaining this stability (Ferri et al., 2007; Kittappa et al., 2007). Further modifications were implemented using new phenotypic markers to show that ventral midbrain DA neurons were not previously obtained, and thereby three modifications were added to the previously established differentiation protocol (Sonntag et al., 2007) in order to generate DA neurons with maintained stable phenotype (Cooper et al., 2010). First, retinoic acid (RA) was added at an early phase and at low dose to improve the regional identity of neural progenitor cells. Second, a high activity form of human SHH was used to permit production of a large population of FOXA2+ neural progenitor cells *in vitro*. Finally, FGF8b was replaced by FGF8a, and WNT1 was added for robust generation of FOXA2+ DA neurons (Cooper et al., 2010).

In another study, iPSCs derived from skin biopsies of patients using either three (OSK) or four (OKSM) lentiviral factors were allowed to commence differentiation into DA neurons via dual SMAD inhibition for 1 day and feeder-free floor plate induction (Devine et al., 2011). Noggin (an inhibitor of BMP4) and SB431542 (an inhibitor of Lefty/Activin/TGFβ pathways) were used for this purpose, in addition to Dorsomorphin (a chemical BMP inhibitor) which acts as a partial substitute for Noggin. This was followed by SHH, WNT1, and Dickkopf WNT signaling pathway inhibitor 1 (Dkk1) blocking antibody treatment for 8 days, then switching the culture conditions to promote maturation of mDA neurons from neural progenitors (Devine et al., 2011).

Earlier in 2009, Cai et al. studied the role of LIM homeobox transcription factor 1a (Lmx1a) in the differentiation of human ESCs into mDA precursor cells *in vitro* and after transplantation into a PD model (Cai et al., 2009). Lmx1a is known to autoregulate and control mDA neurons synergistically with the SHH-FoxA2 pathway (Chung et al., 2009). Results have shown that only Lmx1a-expressing human neuronal progenitor cells have the potential to differentiate into mDA neurons after transplantation into the 6-OHDA rat striatum (Cai et al., 2009). This is of great importance for the development of suitable re-placement tissue for the functional recovery from PD (Cai et al., 2009). Consequently, a complete potential of iPSCs to differentiate into DA neurons is revealed once EB cells, derived from iPSCs transfected by a lentivirus, were forced to express the ventral midbrain determinant Lmx1a, together with DA neuron patterning factor. This resulted in the differentiation of EBs into functional mDA, the cell type mostly affected in PD (Sánchez-Danés et al., 2012).

Human iPSC-derived PD-cell models were later used to have a mechanistic insight into the gene-environmental interaction involved in the pathogenesis of PD, such as the use of small-molecule high-throughput screening to identify new pathways (like MEF2C-PGC1α pathway) as therapeutic targets to combat PD (Ryan et al., 2013). All in all, the main aim behind the use of iPSCs technology in this context remains to be able to convert this new knowledge of PD into effective therapeutic discoveries.

### iPSCs and huntington's disease

Huntington's disease (HD) is an autosomal dominant neurodegenerative disorder caused by a CAG trinucleotide repeat expansion in the huntingtin gene that generates long polyglutamine stretches in the encoded huntingtin protein (HTT). This leads to a massive loss of medium spiny neurons in the striatum and loss of neurons in the cortex as the disease progresses. Personality changes, weight loss, involuntary movements and dementia are the principal changes mostly developed among the people carrying the huntingtin gene mutation.

Transgenic mouse models of HD expressing exon 1 of the human HD gene were developed to mimic the features of the human HD. The R6/2 mouse model has represented the most rapid symptoms with widespread huntingtin inclusions in the brain (Mangiarini et al., 1996; Li et al., 2005; Gil and Rego, 2009). Some therapeutic approaches of neuronal transplantation were analyzed in R6/2 mice aiming to restore dysfunctional neurons. Transplantation of striatal tissue from wild type mice embryos in R6/2 transgenic mice presented a good survival and a well-integrated results within the host brain. However, it was associated with minimal behavioral improvements and no effect on weight loss, which might be due to late transplantation intervention (Dunnett et al., 1998; Gil and Rego, 2009). A more recent protocol combined neural stem cells (NSCs) transplantation with a trehalose enriched diet in R6/2 mice and resulted in more improved motor functions, less aggregations in striatum and extended the lifespan of the animals (Yang and Yu, 2009). This further supports the importance of generating human and patient specific HD-iPS neuron cell models with endogenous CAG expansion to be used for cell replacement therapies as well as for drug screening and to enrich our knowledge in understanding mechanisms of HD (Table 2). The generation of efficient protocols for the differentiation of iPSCs into enriched populations of GABA MS-like neurons (GMSLNs) is indeed needed to provide a good model to investigate the disease manifestation and drug development (Nekrasov et al., 2016). HD-specific iPSCs were first generated in 2008 by Park et al. and they expressed an expanded CAG repeat sequences (72 repeats). The iPSCs were then differentiated into neural precursors by re-suspending colonies in EB differentiation medium in the absence of doxycycline with low-speed shaking (Park et al., 2008a).

**Table 2:** **Huntington's disease modeled with patient-specific hiPSCs**.

**Authors**	**Donor somatic cell used**	**Generated iPSCs used**	**Neuronal differentiation method and results**
Park et al., 2008a	Human dermal fibroblasts	Patient specific HD-iPSCs with 72 CAG repeats in huntingtin gene	Resuspending HD-iPSC colonies in EB differentiation medium in the absence of doxycycline
Zhang et al., 2010	Human dermal fibroblasts	Patient specific HD-iPSCs	Treating HD-NSCs with SHH, DKK1, BDNF and ROCK inhibitor Y27632 for 8–10 days (stage 1), then with BDNF, cAMP, VPA, and Y27632 for an additional 1-3 days (stage 2)
Camnasio et al., 2012	Human skin fibroblasts	Patient specific HD-iPSCs	(Chambers et al., 2009) neural differentiation protocol used revealing that the lengths of CAG trinucleotide repeats in the generated neurons is not affected by the differentiation process

*hiPSCs, human induced pluripotent stem cells; HD, Huntington's disease; EB, embryoid body; SHH, sonic hedgehog; DKK1, Dickkopf WNT signaling pathway inhibitor 1; BDNF, brain-derived neurotrophic factor; VPA, valproic acid; HD-NSCs: HD, specific neural stem cells*.

Later in 2010, Zhang et al. piloted a study to differentiate and characterize human HD cell model from iPSCs (Zhang et al., 2010). Their iPSC cell model had CAG repeats of the same length as the parental fibroblast cells (72 repeats). Neural induction of these HD-iPSCs was achieved using the previously established EB differentiation method (Park et al., 2008a). Thereafter, further differentiation of the HD-specific NSCs (HD-NSCs) into striatal neurons was carried out by treating them directly with SHH, DKK1, brain-derived neurotrophic factor (BDNF) and ROCK inhibitor Y27632 for 8–10 days as an initial step (stage 1), then with BDNF, cAMP, VPA and Y27632 for an additional 1–3 days (stage 2). At stage 1, cells stained positive for the neuronal markers TUJ1 and GABA, as well as calbindin. Mature striatal neurons at stage 2 expressed, in addition to the aforementioned three markers, an additional medium spiny neuron marker DARPP-32, thus confirming their striatal nature (Zhang et al., 2010).

To assess whether the length of the pathological CAG repeat in GABAergic neurons derived from HD-iPSCs is affected during lentiviral reprogramming, a study was conducted showing that neither the long-term growth of reprogrammed HD-iPSCs *in vitro* nor the differentiation process affects the lengths of CAG trinucleotide repeats in these neurons (Camnasio et al., 2012). In this study, the neural differentiation protocol described by Chambers et al. was used (Chambers et al., 2009), where it is shown that the synergistic action of two SMAD signaling pathway inhibitors, Noggin and SB431542, is sufficient to induce rapid and complete neural differentiation. These advances in the use of HD-specific iPSCs and neurons differentiated from them may provide a powerful platform for target identification and drug screening in HD.

### iPSCs and amyotrophic lateral sclerosis

Amyotrophic lateral sclerosis (ALS) is an incurable neurodegenerative disorder that leads to the loss of upper and lower motor neurons. It has a genetic background in which 10% of the cases have a positive family history of mutations in the superoxide dismutase 1 (SOD1) gene which is associated with the most common familial form of ALS. In addition, there is an estimate of 30 genes directly linked to the pathophysiology of the disease, including TARDBP, FUS, OPTN, VCP, UBQLN2, C9ORF72, PFN1…etc., and over 120 other genes indirectly associated with ALS (Abel et al., 2012). Generation of iPSCs from patients with ALS and their differentiation into motor neurons was first reported in 2008 (Dimos et al., 2008; Table 3). In his study, Dimos et al. (2008) used skin fibroblasts collected from an 82-year-old patient diagnosed with familial ALS to produce patient-specific iPSCs. These cells were subsequently plated in suspension culture to form EBs, then treated with RA and recombinant SHH to persuade neural differentiation. When these differentiated EBs were plated on a laminin-coated surface and allowed to mature for 7–15 days, they started forming neuron-like outgrowths that stained positive for TUJ1, confirming their neuronal nature.

**Table 3:** **Amyotrophic lateral sclerosis modeled with patient-specific hiPSCs**.

**Authors**	**Donor somatic cell used**	**Generated iPSCs used**	**Neuronal differentiation method and results**
Dimos et al., 2008	Human skin fibroblasts	Patient-specific iPSCs carrying SOD1 gene mutation (with L144F dominant allele)	Allowing iPSCs to form EBs in suspension culture, then treating them with RA and recombinant SHH to induce neural differentiation, and finally plating them on a laminin-coated surface and culturing for 7–15 days
Chestkov et al., 2014	Human skin fibroblasts	iPS cell lines from patients with SOD1- associated ALS	Adding RA and SHH to mTeSR1 culture medium 12 days after iPSCs generation, then maturation of the produced motor neurons using BDNF and GDNF
Li et al., 2015	Human skin fibroblasts	Familial ALS patient-specific iPSCs, carrying different ALS mutations, including SOD1 and FUS	Differentiation to NPCs by inhibition of SMAD pathway via EB formation assay

*iPSCs, induced pluripotent stem cells; SOD1, superoxide dismutase 1; ALS, Amyotrophic lateral sclerosis; FUS, fused in sarcoma gene; EB, embryoid body; RA, retinoic acid; SHH, sonic hedgehog; BDNF, brain-derived neurotrophic factor; GDNF, glial cell line-derived neurotrophic factor; NPCs, neuroprogenitor cells*.

Recently in 2014, researchers were able to obtain iPSCs from patients with familial forms of SOD1-mediated ALS by using lentiviral reprogramming system (Chestkov et al., 2014). Using a similar method to Dimos et al. (2008) and Chestkov et al. (2014) generated patient specific iPSC cells carrying the SOD1 mutation from primary skin fibroblasts. The resulting iPSCs expressed the same SOD1 gene mutations as the respective patients and no differences were detected among the iPSCs of the different patients with different genotypes. After 12 days, these cells were directly differentiated into motor neurons by adding RA and SHH to the culture medium. Additionally, BDNF and glial cell line-derived neurotrophic factor (GDNF) were used for the maturation of the motor neurons. These cells also expressed TUJ1 neuronal marker (Chestkov et al., 2014) but their advantage over the model of Dimos et al. (2008) was mainly due to the presence of the mTeSR1 in medium that helped in maintaining the pluripotent state needed for the unlimited production of motor neurons (Chestkov et al., 2014). That being said, it had been shown that mTeSR1 medium supports stem cell growth by containing agonists of signaling systems, such as GABA receptors and ErbB2, which while less characterized, are thought to play a role in human ESCs maintenance (The International Stem Cell Consortium Initiative Consortium et al., 2010). Nevertheless, many other media, like DMEM/F12 medium with 20 ng/mL β-FGF (Cai et al., 2009), that has been used for maintaining iPSCs, are also capable of avoiding uncontrolled differentiation of those iPSCs.

Moreover, astroglia were derived from iPSCs obtained from an ALS patient carrying the *TARDBP* mutation in order to investigate the suspected role of glial cell activation in ALS pathogenesis. These derived astroglia showed TDP-43 (the protein product) proteinopathy, such as mislocalization of TDP-43, increased total cellular levels of TDP-43, and decreased cell survival. However, upon co-culture of the derived astroglia with derived motor neurons from the same iPSCs of the ALS patient or from control normal patients, there were no effects of any kind on the neurons in culture suggesting other involved mechanisms as previously described for *SOD1* ALS (Serio et al., 2013). In this context, motor neurons were successfully differentiated from iPSCs of an 82 year old patient with familial ALS of this same TDP-43 mutation (Dimos et al., 2008). Besides, iPSCs have been derived from ALS patients carrying the *C9ORF72* mutation which is also involved in frontotemporal dementia and these have also uncovered some characteristic phenotypes in ALS (Donnelly et al., 2013; Sareen et al., 2013; Hedges et al., 2016). All this further supports the notion of iPSCs' culture studies as models unraveling a lot that needs to be known about neurodegenerative disease such as ALS that we know about the very little.

Although motor neurons have been successfully produced via differentiation of ALS-iPSCs, no reports indicated whether SOD1 mutation interferes with this differentiation. Therefore, Li et al. tackled this issue in 2015 (Li et al., 2015) whereby ALS-iPSCs were derived from fibroblasts using retroviruses. This was followed by differentiation into NPCs via EB formation assay by inhibiting the SMAD pathway. Interestingly, no significant differentiation differences were seen between control and SOD1-iPSCs, suggesting that SOD1 mutation has no obvious effects on neural induction. Moreover, NPCs were treated with fetal bovine serum (FBS) to induce astroglia formation, which successfully expressed the astroglia progenitor marker CD44. It is noteworthy mentioning that generation and maturation of iPS derived-astroglia takes a long time *in vitro*, providing a platform to screen drugs that may be used to enhance astroglia development and maturation (Li et al., 2015). Some late studies have also suggested a role for astrocyte pathogenesis in ALS that also express the mutant SOD1 gene contributing to the death of motor neurons (Di Giorgio et al., 2007; Nagai et al., 2007). This authenticates the importance of iPSCs technology in studying both neuronal and astrocytic cell lineages to uncover the mechanism of action of riluzole and to discover new drugs that might provide a ray of hope for ALS patients.

Up till now, FDA has only approved one drug, riluzole, for ALS which acts by delaying progression of the disease but has no assertive efficacy in increasing survival (Cheah et al., 2010). This authenticates the importance of iPS cell technology in providing a ray of hope for those patients who continue to suffer the lack of an effective remedy for their condition.

### iPSCs and alzheimer's disease

Alzheimer's disease (AD) is the most common form of age-related dementia (Alzheimer's Association, 2010), characterized by progressive cognitive disturbance and loss of memory. It is famously distinguished by the presence of two major hallmarks: extracellular accumulation of amyloid beta (Aβ) plaques and intracellular aggregation of the microtubule associated protein, tau. AD exists in as little as 1–5% in its familial form, characterized by autosomal dominant inheritance of Presenilin-1 or -2 or/and Amyloid Precursor Protein (APP), while the majority of AD cases to date are sporadic and multifactorial with suspected role of epigenetics involved in the course of progression. List of suspected genes include *MAPT, BACE1, BACE2, ADAM10, ADAM17*, and others (Karch and Goate, 2015).

This neurodegenerative disease is under extensive study for therapeutic options including cell-replacement based therapy. The need for urgent therapy stems from the fact that a small percentage of all AD patients get moderate improvement from all available AD drugs and a significant number of those patients suffer major side effects (Serretti et al., 2007). It is consistent that in AD animal models, there is decrease in neurogenesis in the subventricular and subgranular zones that get initiated early before Aβ plaque formation suggesting decreased neurogenesis and progressive neuronal loss as pathologies of AD (Yang et al., 2016).

Transplantation of cholinergic precursors differentiated from iPSCs into AD transgenic mice proved to restore spatial memory impairment and survival of the cells transplanted (Fujiwara et al., 2013). A particular study reported that medial ganglionic eminence-like progenitors obtained from iPSCs differentiation, completed their maturation into the forebrain as GABAergic interneuron subtypes with mature physiological properties within a prolonged period of time that extended until 7 months, thus mimicking endogenous human neural development (Nicholas et al., 2013).

Yet, the difficulty of obtaining live neurons from patients, and the incapacity to pattern the sporadic form of the disease, still represents a limitation in the understanding of AD. Nevertheless, it is now possible to surmount this problem by simply obtaining fibroblast from skin biopsy of these patients and then generating disease specific iPSCs, which would serve as a model to enrich our knowledge in this disease (Table 4).

**Table 4:** **Alzheimer's disease modeled with patient-specific hiPSCs**.

**Authors**	**Donor somatic cell used**	**Generated iPSCs used**	**Neuronal differentiation method and results**
Yagi et al., 2011	Human skin fibroblasts	AD-derived iPS cell lines with PS mutations (PS1 and PS2 iPSCs)	PS mutations in familial AD shown not to affect neuronal differentiation
Yahata et al., 2011	Human dermal fibroblasts	AD-derived iPSCs	Differentiation of hiPSCs into forebrain neurons achieved using protocol described by Chambers et al. (2009; Using two SMAD signaling pathway inhibitors, Noggin and SB431542, for rapid and complete neural differentiation), and additionally induced with Noggin and SB431542 for 17 days
Nieweg et al., 2015	Human cord blood-derived unrestricted somatic stem cells (Zaehres et al., 2010)	hiPSC line 8/25 derived from human cord blood-derived unrestricted somatic stem cells (Zaehres et al., 2010)	hiPSC line 8/25 cultured in mTeSr medium and differentiated into neural cells according to a modified protocol by Li et al. (2009), where a neural medium was used that comprises of N2B27 medium (50% DMEM/F12, 50% Neurobasal), Glutamax, penicillin, streptomycin, modified N2 supplement, β-mercaptoethanol, B27 without vitamin A and heparin

*hiPSCs, human induced pluripotent stem cells; PS, Presenilin; AD, Alzheimer's disease*.

Yagi et al. (2011) and Yahata et al. (2011), were the first to generate hiPSCs from human fibroblasts in 2011. Differentiation of hiPSCs into forebrain neurons was achieved as described previously by Chambers et al. (2009). In addition, NSCs were induced with Noggin and SB431542 for 17 days to obtain cells that stained positive for the neuroectodermal marker, Nestin (Yahata et al., 2011). The presenilin (PS) mutations in familial AD were proved not to affect neuronal differentiation, where the ability to generate neurons (~80% TUJ1-positive cells) was comparable between PS-iPSCs and control iPSCs (Yagi et al., 2011). Noteworthy, the differentiated cells expressed APP, β-secretase, and γ-secretase components, and were found to secrete Aβ into the conditioned media in addition to expressing a glutamatergic phenotype. Another important finding was that neurons differentiated from iPSCs of familial AD patients with PS1 and PS2 mutations exhibited increased production of Aβ-42, and tau protein was found hyper-phosphorylated thus further showing that the differentiated iPSCs truly recapitulate the pathogenesis of AD (Yagi et al., 2011).

In a recent work published in 2015, Nieweg et al. used human iPSC-derived cortical neurons, differentiated using an EB system similar to that applied by Li et al. (2015), to produce a highly reproducible cellular AD model that facilitates the mechanistic analysis of Aβ-induced synaptic pathomechanisms and the development of new therapeutic approaches (Nieweg et al., 2015). The differentiation protocol described by Li et al. states culturing iPSCs in a neural medium comprising Dulbecco's Modified Eagle Medium (DMEM)/F12, N2 supplement and heparin without growth factors (Li et al., 2009). Nieweg et al. (2015) used a modified medium that comprises of N2B27 medium (50% DMEM/F12, 50% Neurobasal), Glutamax, penicillin, streptomycin, modified N2 supplement, β-mercaptoethanol, 1% B27 without vitamin A and heparin. In the same paper, an attempt was made to recapitulate the synaptotoxicity of Aβ which is crucial for understanding the cascade of events leading to cell death and continuous brain degeneration. The cells were differentiated into deep layer cortical pyramidal neurons and GABAergic interneurons; and upon longer cultivation, these cells exhibited action potential generation and excitatory and inhibitory synapses. Yet, most interesting was that these AD-derived neurons were very susceptible to Aβ synaptotoxicity (Nieweg et al., 2015). In a study on epigenetic characterization of iPSCs DA differentiated neurons, there was significant difference in global gene expression and DNA methylation as compared to the *in vivo* DA cells (Roessler et al., 2014). Therefore, epigenetic changes seem to leave their mark on the genome even beyond de-differentiation and this is mainly considered a limitation for applying iPSCs in human therapies. Since the cells in these experiments were needed to be passaged and tested at several time points, trans-differentiation does not seem to be the proper method for such application. Yet, all these models offer valuable insight about AD and understanding its progression and further designing therapeutics.

## iPSCs and spinal cord injuries

In case of injury, the spinal cord does not spontaneously regenerate itself and till now there is not one available treatment that can provide the least functional recovery from SCI. The level at which the injury is present determines the symptoms and consequent motor manifestations. To date, cervical SCIs account for the majority of presented SCI cases (Doulames and Plant, 2016).

Many previous cell transplantation therapies have been approached such as peripheral nerve bridges, Schwann cells, olfactory glia, mesenchymal stem cells and NSCs (Plant et al., 2001; Barry and Murphy, 2004; Ramer et al., 2004; Cummings et al., 2005; Parr et al., 2008). Such therapies function to ameliorate the existing damage and prevent its further exacerbation by providing a scaffold to bridge the lesion, replace the host dead or lost neurons or glia, promote axonal regeneration and overcome glial scar formation (Bregman et al., 1997; Jones et al., 2001; Raisman, 2001; Ikegami et al., 2005; Donnelly and Popovich, 2008). In SCI, the main drawback to regeneration is the inability of the CNS neurons to regenerate axons that can cross through the inhibitory milieu of the glial scar and the injured lesion (Horner and Gage, 2000). Certain studies on rats showed promise that this can be overcome by using iPSCs as these reprogrammed iPSC neurons can extend many axons over very long distances and form synapses with host rat neurons (Parr et al., 2008; Romanyuk et al., 2015).

The implementation of iPSCs offers a great mean of cell-based therapy capable of bypassing the usual ethical dilemma associated with ESCs. The iPSCs have the ability to differentiate into tissue-specific neurons which leads to long-term restoration of the lesioned tissue (Romanyuk et al., 2015). Moreover, these cells can be obtained in a non-invasive patient-specific manner that makes iPSC an even more approachable attractive candidate for SCI therapy. This cell replacement therapeutic approach has opened a new era in the field of regenerative medicine.

Jin Young Hong and colleagues generated self-renewable induced NSCs from somatic fibroblasts and engrafted them in a rat model of SCI. The engrafted cells were able to restore axonal regeneration resulting in recovery of motor, sensory and autonomic functions (Hong et al., 2014). In another study, Pajer et al. performed avulsion of the lumbar 4 (L4) ventral root in rats, an injury that is known to induce the death of majority of affected motor neurons. Afterwards, they transplanted murine iPSCs into the injured spinal cord segment. Their observation included improved re-innervation by the host motor neurons as compared to controls with no iPSC transplantation procedure. It also seemed that the observed morphological re-innervation resulted in functional recovery as the grafted rats exhibited more motor movement units in their re-innervated limb than controls. This study also established that the grafting of iPSCs downregulated astroglial activation in the injured site and was able to conclude that the observed motor neuron survival and regeneration came as a result of neurotrophic and cytokine modulatory mechanisms (Pajer et al., 2015). Furthermore, a study performed on mice suggested that the neurons derived from the transplanted cells functioned as interneurons in the mouse spinal cord which in turn contributed to the reconstruction of neural circuits (Nakamura and Okano, 2013). Moreover, neural precursors derived from a clone of hiPSCs (IMR90) were used to treat a rat spinal cord lesion 1 week after induction. These hiPSC-neural precursors robustly survived in the lesion, migrated, and partially filled the lesion cavity during the entire period of observation. Transplanted animals displayed significant motor improvement already from the second week after the transplantation (Romanyuk et al., 2015).

The application of iPSCs seems so good so far and all these mentioned results raise great clinical expectations. However, it should be noted that safety-related concerns for such iPSCs cell therapy should be resolved prior to clinical application. A main concern after iPSC therapy is tumor formation as a result of residual or remaining undifferentiated iPSCs that were not successfully induced into differentiation. Moreover, there is the chance that the reprogramming was not necessarily complete (Nakamura and Okano, 2013; Kim et al., 2014). Long term safety issues also include deteriorated motor function accompanied by a tumor formation (Nori et al., 2015). However, Tsuji et al. used the neurospheres 3D culturing of iPSCs obtained from mice fibroblasts, and injected them into the injured spinal cord. Those neurospheres were able to differentiate into three neuronal lineages: astrocytes, oligodendrocytes, and neurons, promoting recovery and improving locomotor functional loss with no tumor formation for the observation period of more than 120 days (Tsuji et al., 2010). Besides, an *in vivo* study has shown that 3 rats have died out of 12 rats that received transplantation with DA neurons derived from protein based iPSCs (Rhee et al., 2011). The rats that died showed tumor formation after 8 weeks from grafting. Moreover, in a comparison between transplanted secondary neurospheres derived from iPSCs generated in 11 different ways and neurospheres from ESCs, the former showed a significant teratoma formation propensity most likely correlated with the persistence of undifferentiated cells (Miura et al., 2009). All this further halts the use of such a therapeutic tool in humans as much still needs to be optimized.

Researchers at the University of California, San Diego School of Medicine launched a 5-year clinical trial program starting 2014 in order to investigate the safety of NSCs transplantation in patients with chronic SCIs (University of California 2014). A recent clinical trial on humans, the SciStar study, has been using oligodendrocyte progenitors to treat people with recent SCIs. This study not only has proven to be effective so far but has lately passed major safety issues and therefore was approved to expand by increasing the number of both enrolled patients and the transplanted cells per patient (California Institute for Regenerative Medicine (CIRM) 2016). Although this study aims at evaluating the safety and effectiveness of AST-OPC1 agent, which consists of oligodendrocyte progenitor cells produced from human ESCs, in patients with recent SCI, results can be promising as regards potential use of iPSCs instead of human ESCs in this context.

## Stroke, brain injury and iPSCs

Oki et al. (2012) provided the first evidence that transplantation of hiPSC-derived cells is a safe and efficient approach to promote recovery after stroke and can be used to supply the injured brain with new neurons for replacement. They transplanted neuroepithelial-like stem cells, generated from adult human fibroblast-derived iPSCs, into stroke-damaged mouse and rat striatum or cortex. The transplanted cells stopped proliferating after a while but have at least shown that they can survive without forming tumors for at least a period of 4 months. This 4-months observation period warrants lack of rejection of the transplanted cells and creates an optimal setting to evaluate tumorigenicity. The iPSCs successfully differentiated neurons in intrastriatal grafts sent axonal projections to the globus pallidus. Moreover, the grafted cells exhibited electrophysiological properties of mature neurons and most importantly received synaptic input from host neurons (Oki et al., 2012). There is not much data and research on the iPSCs regenerative ability in brain injury. Therefore, in order to establish its safety and effectiveness, much more studies and effort have to be done in that domain. After all, transforming such data to clinical trials in humans would be a great achievement toward finding a treatment and getting more insight on the brain circuitry itself.

## Limitations of iPSCs use in neurodegenerative diseases

While the iPSCs technology holds the promise to becoming an efficient therapy for many neurodegenerative diseases that currently have no cure, there remains the risk of encountering multiple unanticipated outcomes when applying them on humans. The risks range from unwanted biological effects and immune response, toxicity, neoplasm formation, disease transmission, reactivation of latent viruses, to rejection of the cells by the body. It is difficult to determine and pinpoint the risks as they depend on several factors, including the cells that are used to achieve pluripotency, the status of the differentiated cells, their proliferation capacity, the technique of administration of the pluripotency genes, the level of manipulation, the growth factors used, the dilemma of retaining epigenetic memory, the intended site of injection, the reversibility or even irreversibility of the applied treatment, the susceptibility of the administered cells for disease, the incomplete suppression of the four transgenes after differentiation, the persistence of undifferentiated cells, and the survival of the transplanted cells *in vivo* (Okita et al., 2007). The known risks so far that were obtained mostly from animal models include tumor formation, unwanted immune responses and the transmission of certain adventitious agents (Herberts et al., 2011; Okano et al., 2013). Unfortunately, an estimated 20% of mice that received iPSCs were found to develop tumors (Abdullah et al., 2012). Furthermore, it has been hypothesized that the sustained expression of the transgenes might have the ability to change the expression of certain oncogenes or tumor suppressor genes thus altering the tumorigenic potential of the cells. The c-Myc is a risk factor by itself as it is upregulated in naturally occurring tumors.

An important risk of iPSC therapy is associated with using lentiviruses or retroviruses. These viruses, although are genetically tailored to hold the genes required for an iPSC state transformation, will integrate into the host cell genome and consequently add multiple viral integration sites and be the cause for several safety issues (Howe et al., 2008). However, it should be noted that much control was gained over this process as the viral integration site can be determined in iPSCs using Cre-mediated strategies for instance or by using adenoviruses, plasmids, transposons, recombinant proteins, Sendai virus vectors and modified RNA (Herberts et al., 2011). It should be further noted that there has been worries of a state of dedifferentiation or dedifferentiation into an unwanted cell type once transplanted to humans, though this remains clinically unclear. Therefore, there has to be a thorough consideration of all the suspected risk factors before setting into iPSCs human clinical trials.

Trans-differentiated neurons offer a way to bypass the tumorigenicity risk associated with passing through a pluripotency state. This method allows lineage reprogramming as in directly converting a somatic cell type into another through transgenic expression of transcription factors or miRNAs. On the other hand, the procedure of re-differentiating iPSCs into specific cell types is considered lengthy, costly and arduous. In addition to that, having multiple stages before reaching the intended outcome may lower the efficiency of the generated cell type. So far, in this context, trans-differentiation seems to be also an interesting path in research that also offers the same concept of therapeutic approaches and disease modeling as iPSCs. Trans-differentiated cells show no tumorigenicity when transplanted *in vivo*, plus these cells show similar functionality to cells derived from iPSCs (Lopez-Leon et al., 2014; Hou and Lu, 2016). In the presence of an insult or a lesion, or even in AD animal models, glial cells can be successfully transformed to functional neurons by single transcription factor intervention (Guo et al., 2014). For example, in the presence of SOX2 in the injured adult spinal cord, astrocytes convert to double cortin (DCX) positive neuroblasts (Lau et al., 2014). However, the major limitation for trans-differentiation to be a therapeutic approach is that the obtained population of induced neurons has little to no proliferation rate, therefore directly restricting the efficiency and expansion of this technique (Hou and Lu, 2016). Hence, a better investment for a drug or therapeutic or modeling purposes would still be iPSCs, but this does not overthrow the importance of trans-differentiation. The research in this field is still young but so vivid and fertile that we can witness iPSCs applied in neurodegenerative diseases treatments in not the so far future. Yet, until this day, there is no recorded iPSCs treatment application on humans (Trounson and DeWitt, 2016).

## Conclusion and perspectives

Although, iPSCs research has been a revolution in the scientific field as it provides new hope for the treatment of many diseases, protocols describing the differentiation of iPSCs into neural cells in neurodegenerative diseases and in the context of neurotrauma are still being modified and studied to assess the effect of this process on gene mutations in these cells and vice versa. Advances have been made recently to uncover the underlying molecular pathways of several neurodegenerative diseases, yet more work has to be done before one can say complete cure from such disorders is possible.

A recent approach of generating 3D brain tissues, namely “cerebral organoids”, closely reproduces the endogenous developmental program. This approach can give rise to retinal identities, ventral telencephalon, developing cerebral cortex, and choroid plexus, within 1–2 months (Lancaster and Knoblich, 2014). Recent development of these 3D brain organoids derived from human iPSCs is a promising technology for understanding the development of *in vitro* disease models and investigating in particular the human polygenic disorders where animal models are not sufficient (Lindborg et al., 2016; Quadrato et al., 2016).

**Figure 2:**
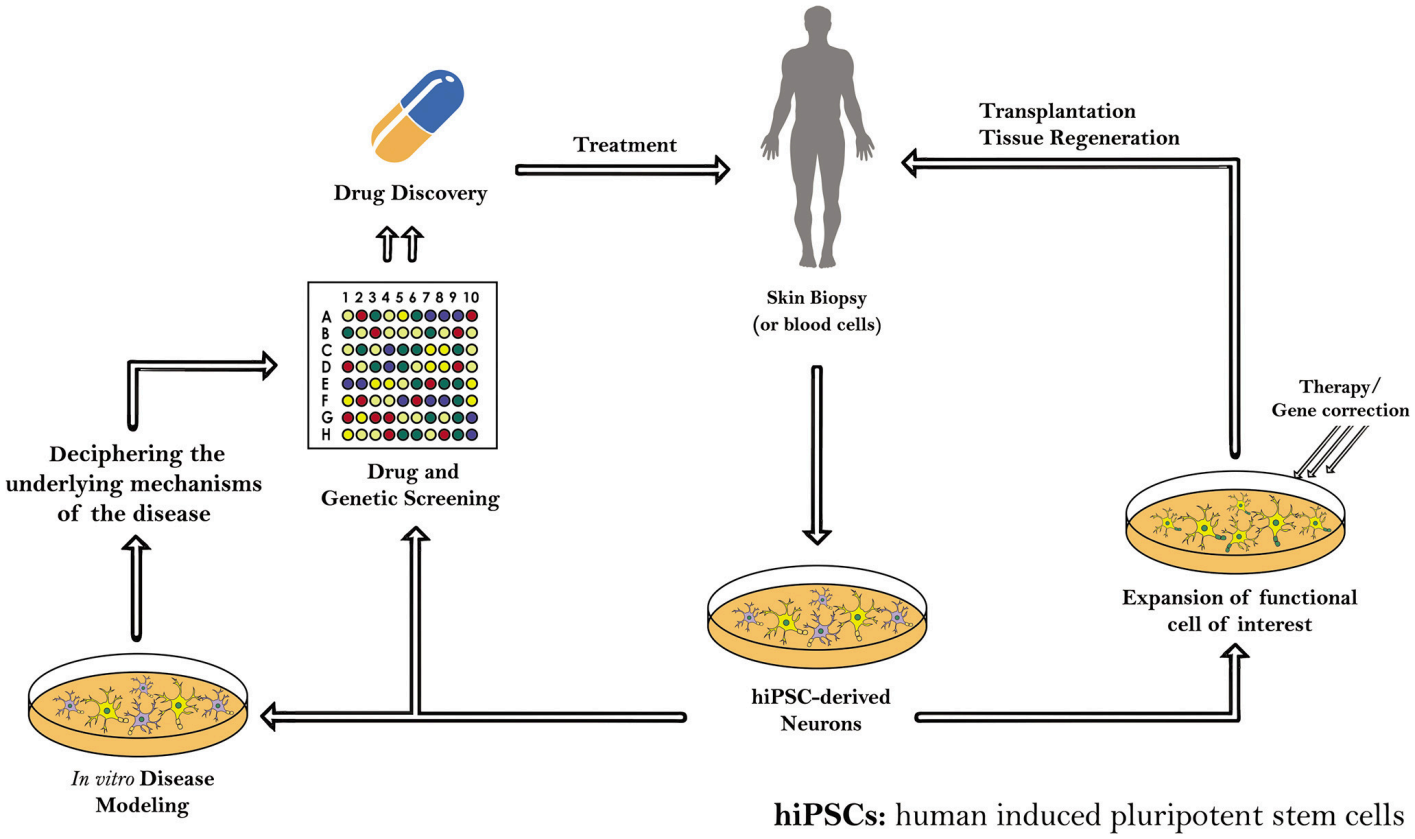
**Schematic diagram demonstrating the different applications of induced pluripotent stem cells (iPSCs) derived from human somatic cells**. The patient-specific hiPSCs and hiPS-derived neurons can serve as precursors for transplantation and tissue regeneration therapy. hiPSCs generated are also a copious resource for *in vitro* and *in vivo* disease modeling, drug and genetic screening, and regenerative medicine.

Yet, many hitches like mutations, incomplete epigenetic reprogramming and tumors formation, which accompany the use of iPSCs, should be solved as well. Therefore, further understanding of iPSCs, including a genome-wide epigenetic characterization of those cells and further studying of their genomic stability, is needed before beginning their clinical applications in the area of regenerative medicine for treating human diseases, mainly the intractable ones (Table 5). Indeed, these patient-specific hiPSCs will serve in the future as precursors for transplantation and tissue regeneration therapy, as well as a copious resource for *in vitro* and *in vivo* disease modeling and drug and genetic screening (Figure 2).

**Table 5:** **Different outcomes of iPSCs differentiation in the different diseases and properties of the cells obtained**.

**Disease**	**Type of differentiated neurons from iPSCs**	**Characteristics of iPSCs**	**Most common characteristics of the differentiated cells**	**Cell Physiology & Morphology**	**Cell survival**	**Transplantation in animal models**
PD	Dopaminergic neurons	Similar Morphology to ESCs Morphological, proliferative, and clonogenic characteristics(patient derived) very similar to naive mouse ESCs (Hu et al., 2015)	β3-tubulin+, TH+, VMAT2+, NURR1+, GIRK2+, MAP2+, Nestin+, Foxa-2+, Lma1+ (Wenker et al., 2015)	Increased glucosylceramide and α-synuclein, Tau, MAPT Alterations of autophagic and lysosomal mechanisms Dysregulation of calcium homeostasis Altered Morphology Increased ROS and Oxidative stress (Wenker et al., 2015)	Vulnerable	Ameliorate or improve motor symptoms of PD - teratoma formation *in vivo* & glial and neuronal differentiation and integration into pre-existing networks *in vivo* as well (Wernig et al., 2008) Spontaneous differentiation *in vitro*
AD	Cholinergic neurons, glutamatergic and other neurons	Apoptotic loss upon extended culturing especially with extrinsic Zic1 expression (Qiang et al., 2011) High efficiency differentiation to neurons (90%) from familial AD iPSCs (Israel et al., 2012)	NeuN+, Tau+, NCAM+, MAP2+, double vGLUT1+ AND MAP2 + (Qiang et al., 2011) Gene expression profiling more similar to CNS neurons than to fibroblasts, astrocytes, astrocytes, neural progenitors, iPSCs or ESCs	Altered processing and localization of APP Increased production of Aβ Typical neuronal Na^+^, K^+^, and Ca^2+^channel properties and functions Ca^2+^channel properties and functions Fire action potential in response to depolarizing injections -Exhibit normal electrophysiology (Israel et al., 2012) -normal resting potential, passive membrane properties and synaptic vesical release (Qiang et al., 2011) Exhibit phenotypes of familial AD samples	More susceptible to glutamate-mediated cell death (Duan, 2014) -Apoptotic loss depending on method of Apoptotic loss depending on method of differentiation and of obtaining induced pluripotency	Improve cognitive function and spatial memory in animal models
HD	Striatal neurons (striatal medium spiny neurons (MSN))	iPSCs and precursor cells show the same CAG mutation expansion as that from the HD patient whom the iPS cell line was established from (reference 44 in the original paper) -HD-NSCs showed enhanced caspase activity when deprived from growth factors compared to normal-NSCs -Higher lysosomal activity in HD-iPSCs	TUJ1+, GABA+, Calbindin+ and -Mature cells express DARPP-32+ (reference 44 in the original paper)	A lot of variables in differentiation efficiency and role of epigenetics traces or memory (Tousley and Kegel-Gleason, 2016) Neuronal (Tousley and Kegel-Gleason, 2016) Neuronal differentiation is reduced in HD-iPSCs vs. normal iPSCs Cells exhibit action potentials with features characteristic of immature cells It is not well well clear how the iPSCs derived MSN are similar to native MSNs in the human brain (Kaye and Finkbeiner, 2013)	–	–
ALS	Oligo-dendrocytes, motor neurons (upper and lower)		Co-expression of LIM homeodomain (LIM-HD) transcription factors: insulin gene enhancer 1 (ISL1), LIM homeobox 3 (LHX3), and pancreas homeobox 1 (Sances et al., 2016)	Non impaired and active maturation No significant or consistent variation in proliferation rates from control normal derived iPSCs No impairment in passive cell properties (Livesey, 2016) ALS iPSCs derived neurons develop voltage-gated K^+^and Na^+^ion channel currents sufficient to generate induced action potentials (early in differentiation) With maturation, action potentials become sharper as Na^+^and K^+^currents improve Very few human iPSCs from ALS can fire trains of action potentials like adult neurons *in vivo* Lower membrane capacitance, inadequate polarized resting membrane potential, and input resistance -lower ability to form synapses (Sances et al., 2016)	Maintain viability (no difference from control)	–
SCI	Oligodendrocytes progenitors or precursor cells, neuronal precursors, NSCs and neurons	Can differentiate to oligodendrocytes, and neurons *in vitro* (also *in vivo* but not directly after injury) No tumorigenesis when transplanted and observed over long periods -Present normal karyotype Do not present nestin after transplantation confirming loss of stem cell identity	NeuN+ neurons, GFap+ astrocytes, 0–4 +oligodendrocytes	Differentiated cells were either GABAergic, glutamatergic, or cholinergic	Maintain viability	Functional recovery in rats: recovery of motor, sensory, autonomic and electrophysiological functions Improved re-enervation by host motor neurons -motor neuron regeneration and survival Form synapses with host neurons -downregulation of astroglial activation at site of injury and reduced inflammatory response Reduced apoptosis and enhanced angiogenesis in injured inflammatory response Reduced apoptosis and enhanced angiogenesis in injured areas (Tsuji et al., 2010)

## Author contributions

HB, OH, FC, and WA worked on study conception and design. HB, OH, FC, KC, BD, and AM screened titles for relevance and abstracted the data from the eligible full text articles. HB, OH, and WA analyzed and interpreted the data. HB, OH, FC, and WA drafted the manuscript. HB, OH, and WA critically revised the manuscript with input from the entire team. All authors have read and approved the final draft.

### Conflict of interest statement

The authors declare that the research was conducted in the absence of any commercial or financial relationships that could be construed as a potential conflict of interest.

## Abbreviations

AD: Alzheimer's disease
ALS: Amyotrophic lateral sclerosis
Aβ: amyloid beta
BDNF: brain-derived neurotrophic factor
BMP: bone morphogenic protein
CRISPR: Clustered Regularly Interspaced Short Palindromic Repeats
DA: dopaminergic
DKK1: Dickkopf WNT signaling pathway inhibitor 1
DM: diabetes mellitus
EBs: embryoid bodies
ESCs: embryonic stem cells
GDNF: glial cell line-derived neurotrophic factor
HD: Huntington's disease
HD-NSCs: Huntington's disease specific neural stem cells
hiPSCs: human induced pluripotent stem cells
iPSCs: induced pluripotent stem cells
ICM: inner cell mass
Lmx1a: LIM homeobox transcription factor 1a
mDA: midbrain dopaminergic
NSCs: neural stem cells
NPCs: neuroprogenitor cells
PD: Parkinson's disease
RA: retinoic acid
SHH: sonic hedgehog
SOD1: superoxide dismutase 1 gene
SCI: spinal cord injury
TUJ1: class III-β-tubulin
VPA: valproic acid.
